# Outcome of illuminated microcatheter-assisted circumferential trabeculotomy following failed angle surgery in *PAX6* aniridic glaucoma: a case report and literature review

**DOI:** 10.1186/s12886-024-03425-6

**Published:** 2024-04-09

**Authors:** Tingyi Wu, Cui Cui, Yuanting Li, Ying Hong, Chun Zhang

**Affiliations:** 1 Department of Ophthalmology, Peking University Third Hospital, 49 North Garden Road, Haidian District, Beijing, 100191 China; 2 Beijing Key Laboratory of Restoration of Damaged Ocular nerve, Peking University Third Hospital, Beijing, China; 3 Department of Ophthalmology, Handan Central Hospital, Handan, Hebei China

**Keywords:** Aniridia, *PAX6*, Aniridic glaucoma, Illuminated microcatheter-assisted circumferential trabeculotomy, Case report

## Abstract

**Background:**

Aniridia is a rare eye disorder with a high incidence of glaucoma, and surgical intervention is often needed to control the intraocular pressure (IOP). Here, we reported a case of illuminated microcatheter-assisted circumferential trabeculotomy (MAT) performed on an aniridic glaucoma patient following a previous failed angle surgery. The surgical procedures for aniridic glaucoma were also reviewed.

**Case presentation:**

A 21-year-old man, diagnosed with aniridic glaucoma, came to our hospital consulting for the poor control of left eye’s IOP despite receiving goniotomy surgery 3 years ago. The IOP was 26 mmHg with maximum topical antiglaucoma eyedrops. The central cornea was opaque and the majority of iris was absent. The gonioscopy and ultrasound biomicroscopy (UBM) demonstrated that 360° anterior chamber angle was closed. The whole exome sequencing of peripheral blood confirmed a 13.39 Mb copy number loss at chromosome 11p15.1p13, containing *PAX6* and *WT1* gene. The 360° MAT surgery was performed on his left eye. At 1-year follow-up, the IOP was 19mmHg with 2 kinds of topical antiglaucoma medications, and the postoperative UBM demonstrated the successful incision of the anterior chamber angle.

**Conclusions:**

The case presented here exhibited a case of aniridic glaucoma treated by MAT surgery. The MAT surgery may be an effective option for IOP control in aniridic glaucoma patients following a previous failed angle surgery.

## Background

Aniridia is a rare congenital panocular disorder with an estimated incidence of 1.6 ~ 2 cases per 100,000 persons, and it is usually caused by mutations in the paired box-containing mammalian gene 6 (*PAX6*) [1]. The *PAX6* gene is located on chromosome 11p13, and its loss of function mutation (haploinsufficiency) is responsible for about 90% of aniridia cases [2, 3]. These cases are often complicated with several ocular abnormalities such as cataract, glaucoma, keratopathy and foveal hypoplasia [4]. Additionally, aniridia may present with systemic anomalies including Wilms tumor, genitourinary anomalies and mental retardation (WAGR syndrome) [5], as deletions of 11p13 may encompass the Wilms tumor predisposition gene (*WT1*) which is located 700 kb beside the *PAX6* [6].

The incidence of glaucoma has been thought to occur in about 50% aniridia cases [7]. The development of glaucoma is significantly correlated with the progressing abnormality of anterior chamber angle [8]. The peripheral rudimentary iris stump gradually migrates anteriorly and synechiae is formed between the abnormal iris tissue and the angle wall. This will finally block the trabecular meshwork resulting in the closure of anterior chamber angle and elevation of intraocular pressure (IOP) [2, 7, 8]. The management of aniridic glaucoma is a quite difficult problem. Topical antiglaucoma medications are initially used to lower IOP, but single use of topical medications will gradually fail, and surgical interventions are needed [2, 7]. Various attempts have been made previously, including goniosurgeries, trabeculectomy, glaucoma drainage device implantation and cyclodestructive procedures [7].

Recently, illuminated microcatheter-assisted circumferential trabeculotomy (MAT) has emerged in the treatment of glaucoma [9]. MAT possesses greater advantages than conventional and 6 − 0 suture trabeculotomy in that it reduces the chance of creating false passages and causing inadvertent tissue dialysis. It also enables surgeons to perform circumferential trabeculotomy with a single incision [10, 11]. According to Adachi’s study, trabeculotomy showed good efficacy in treating aniridic glaucoma [12]. With the help of illuminated catheter, doctors are able to perform trabeculotomy more precisely and in wider range. Based on these theories, we attempted to apply MAT to the treatment of aniridic glaucoma, which has never been reported before to the best of our knowledge. Here, we describe a case of MAT surgery performed in an aniridic glaucoma patient with previously failed goniotomy, and the follow-up period continues for 1 year.

## Case presentation

**Fig. 1:**
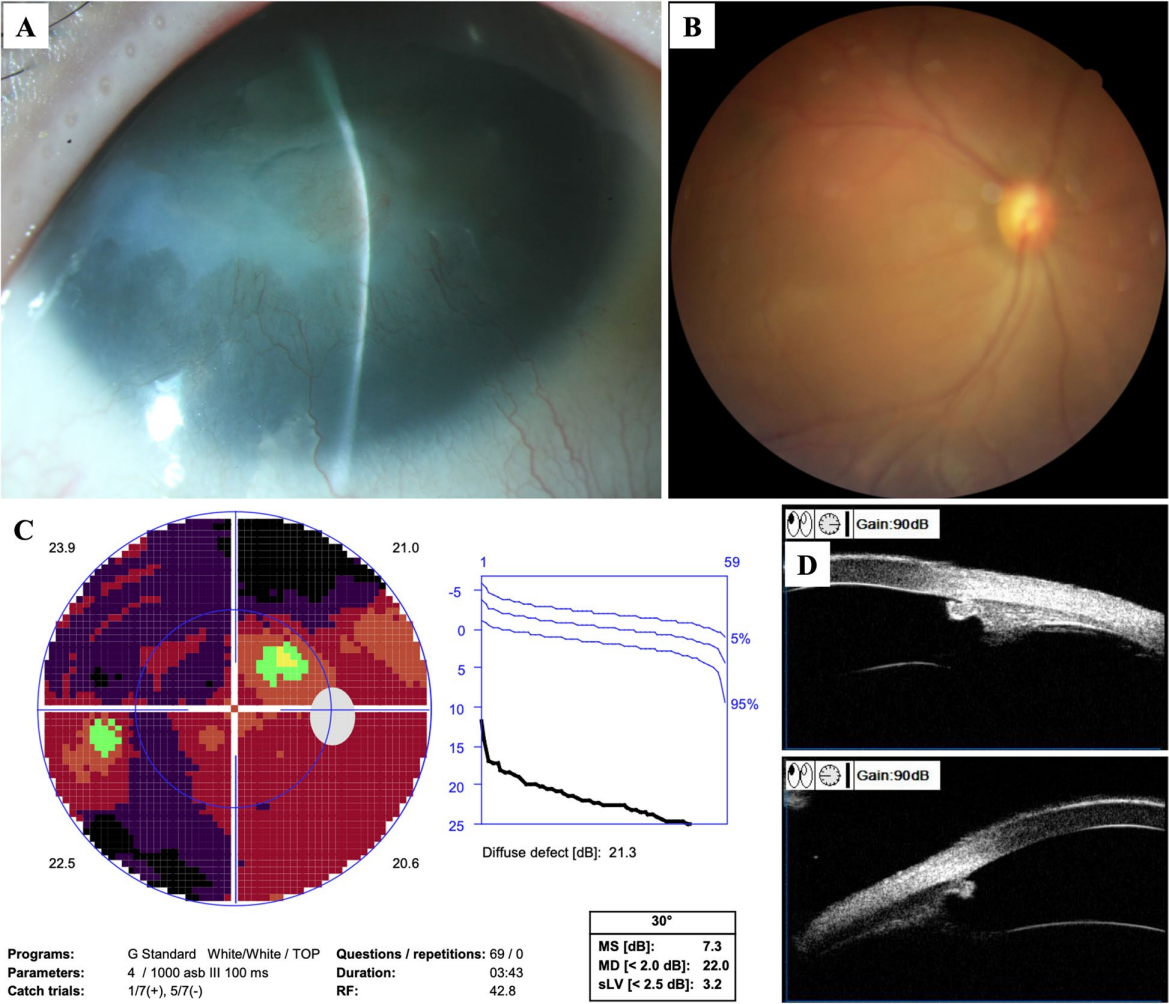
Preoperative examinations of the right eye. **A** The anterior segment photo showed aniridia-associated keratopathy. **B** The fundus photo showed hypoplasia of optic disc and fovea. **C** The static perimetry showed diffuse defect of visual field. **D** The ultrasound biomicroscopy showed the absence of iris tissue and closure of anterior chamber angle

**Fig. 2:**
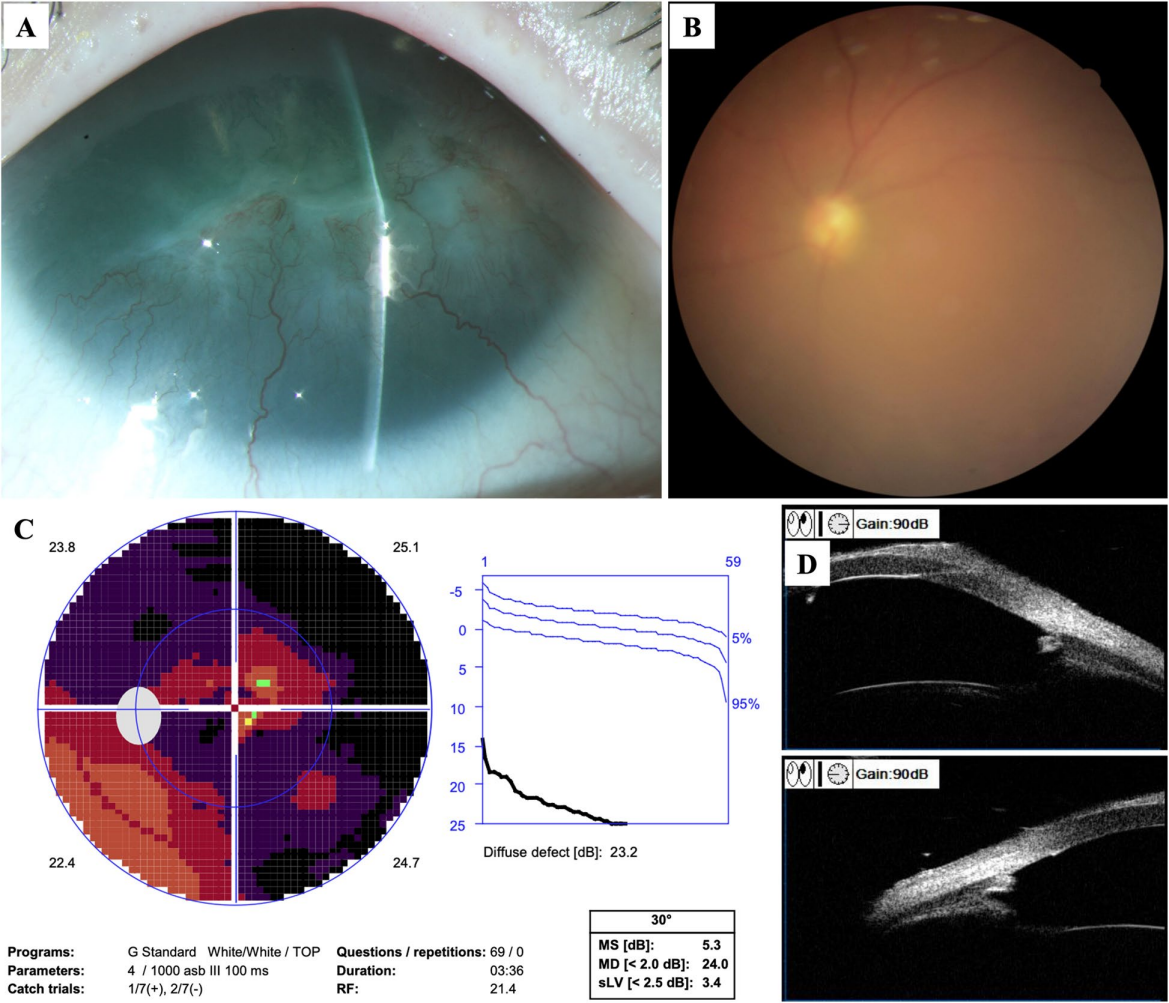
Preoperative examinations of the left eye. **A** The anterior segment photo showed aniridia-associated keratopathy. **B** The fundus photo showed hypoplasia of optic disc and fovea. **C** The static perimetry showed diffuse defect of visual field. **D** The ultrasound biomicroscopy showed the absence of iris tissue and closure of anterior chamber angle

**Fig. 3:**
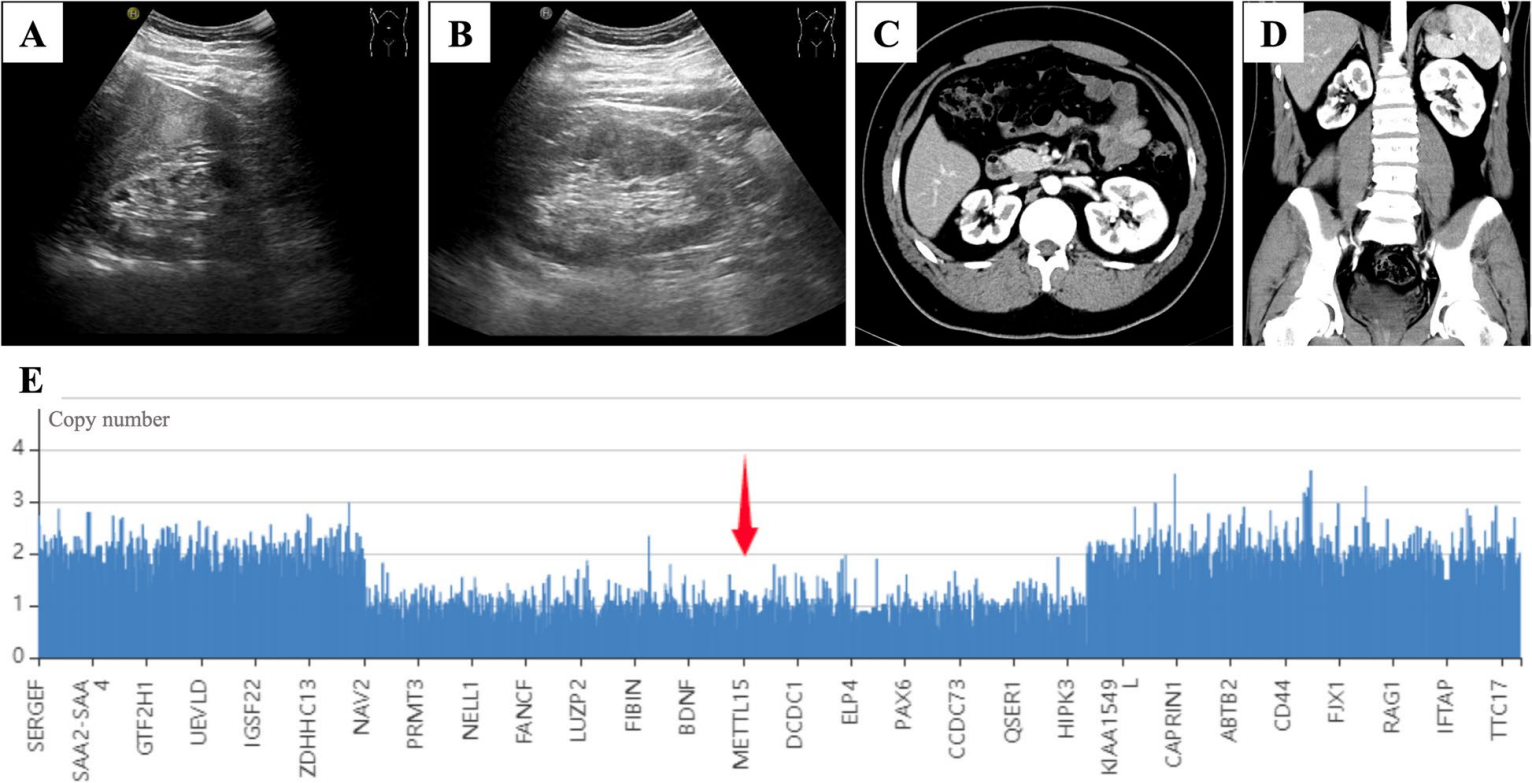
Preoperative examinations of the kidneys and the whole exome sequencing of peripheral blood. **A** The B-scan showed hypoplasia of the right kidney. **B** The B-scan showed a normal left kidney. **C** and **D** The computerized tomography urogram showed hypoplasia of the right kidney and a normal left kidney. **E** The whole exome sequencing of peripheral blood confirmed a 13.39 Mb copy number loss at chromosome 11p15.1p13

A 21-year-old man was admitted to our hospital due to the poor control of left eye’s IOP. Diagnosed with aniridic glaucoma, he had been given topical anti-glaucoma medications to control IOP since the age of one. Three years ago, he underwent goniotomy surgery of the left eye due to the failure of medical therapy, which was a combination of brinzolamide/timolol (AZARGA®, Alcon®, Vernier, Switzerland), latanoprost (XALATAN®, Pfizer, New York City, USA), brimonidine (ALPHAGAN®, Allergan, Dubin, Ireland) and pilocarpine (Bausch & Lomb, Shandong, China). The surgery was accomplished with the help of direct gonioscopy, and the range of goniotomy was 8 ~ 12 o’clock. The IOP was maintained between 16 ~ 20 mmHg after the surgery. He was regularly followed up, and the IOP raised again a month ago. The patient’s best corrected visual acuity was 0.8 OD and 1.0 OS (logMAR). The IOP measured by hand-held rebound tonometry (iCare TA01i tonometer, Vantaa, Finland) was 20 mmHg OD and 26 mmHg OS with brinzolamide/timolol, latanoprost and brimonidine for both eyes. Horizontal nystagmus was present (OU). Slit-lamp examination (OU) showed corneal conjunctivalization and vascularization. The central cornea was opaque and thickened, reflecting a Grade 4 aniridia-associated keratopathy [4]. The majority of iris tissue was absent with peripheral stump remaining, and posterior subcapsular cataract was present. The gonioscopy demonstrated that 360° anterior chamber angle was closed. Fundus examination showed pallor of optic disc (OS). The cup-to-disc ratio was 0.6 OD and 0.8 OS. Foveal reflex was absent, and retinal vessels were seen traversing the expected foveal area (OU) reflecting foveal hypoplasia (Figs. 1 and 2). The patient received cryptorchidism surgery in his childhood. The family history was unremarkable. The computerized tomography urogram demonstrated atrophy of partial renal parenchyma. The whole exome sequencing of peripheral blood confirmed a 13.39 Mb copy number loss at chromosome 11p15.1p13, in which contains *PAX6* and *WT1* gene (Fig. 3).

**Fig. 4:**
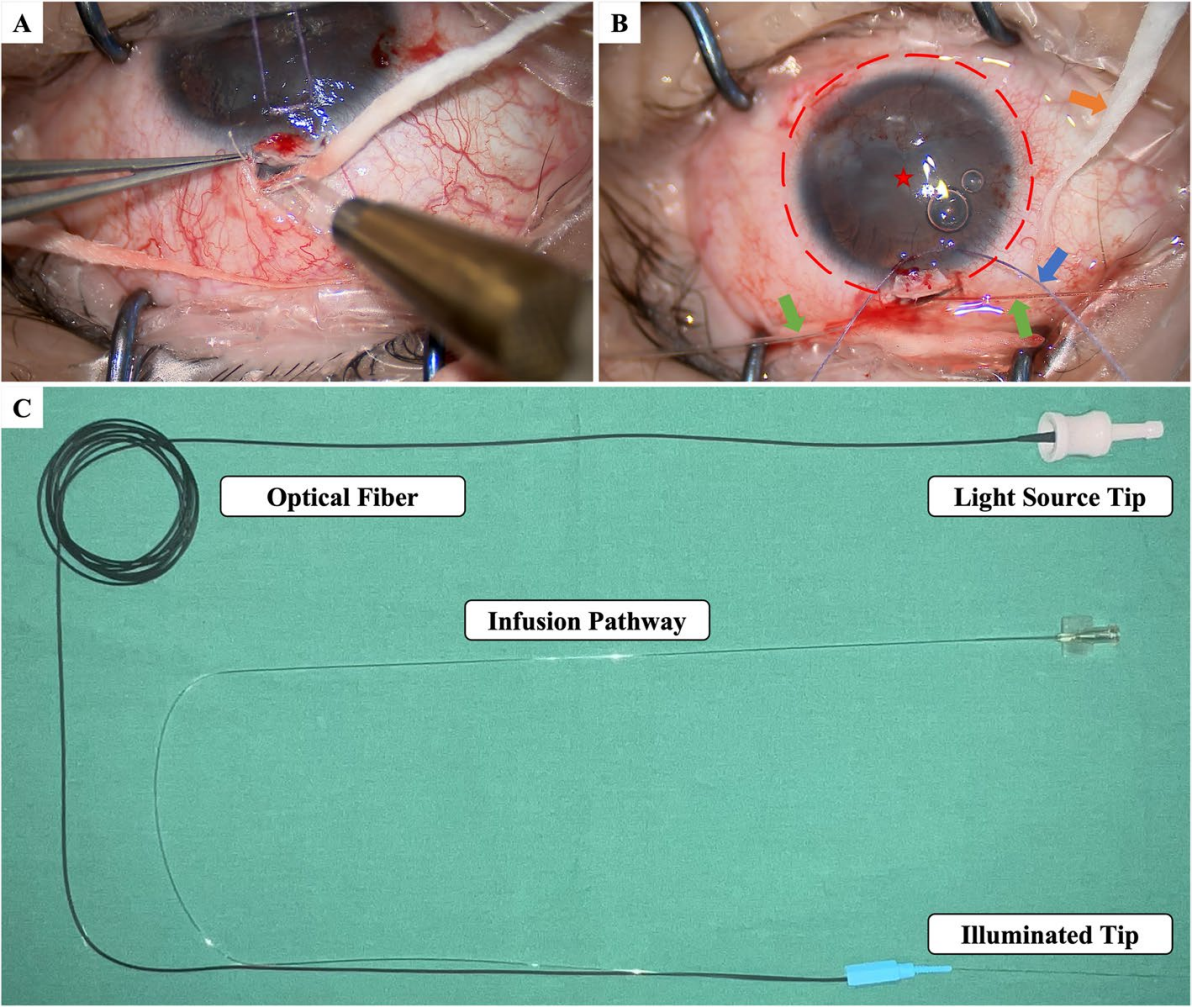
Intraoperative pictures and the image of iTrack™. **A** The scleral flap was made at 12 o’clock. **B** The microcatheter passed through the Schlemm canal circumferentially and came out from the initial breach (red dashed line). Green arrow: the iTrack™ microcatheter. Blue arrow: traction suture. Orange arrow: cotton. Red star: cornea. **C** The appearance of iTrack™

The surgery was performed on the left eye under general anesthesia. Briefly, A fornix-based conjunctival flap was made at 12 o’clock. A 5 $\times$ 4 $\times$ 3-mm trapezoid superficial scleral flap was made at the same location followed by creation of a 2 $\times$ 2-mm deep scleral flap to search for the external wall of the Schlemm canal. Paracentesis was made and viscoelastic was injected into the anterior chamber. A microcatheter (iTrack™, Nova Eye Medical Limited, Kent Town, Australia) was introduced via a breach of the Schlemm canal, and it passed through the Schlemm canal under the guidance of indicator at the tip of the microcatheter. 360° trabeculotomy was accomplished by pulling the two ends of the catheter. 10 − 0 nylon sutures were used to close the scleral flap and conjunctival flap. The anterior chamber was then irrigated from the paracentesis, followed by stromal hydration for corneal incision sealing. The intraoperative pictures and the image of iTrack™ are shown in Fig. 4. After the surgery, levofloxacin eyedrop QID (Cravit^®^, Santen, Osaka, Japan) and prednisolone acetate eyedrop QID (Pred Forte^®^, Allergan, AbbVie Inc., North Chicago, IL, USA) were given for 1 month, and pilocarpine eyedrop QN was given for 3 months.

**Fig. 5:**
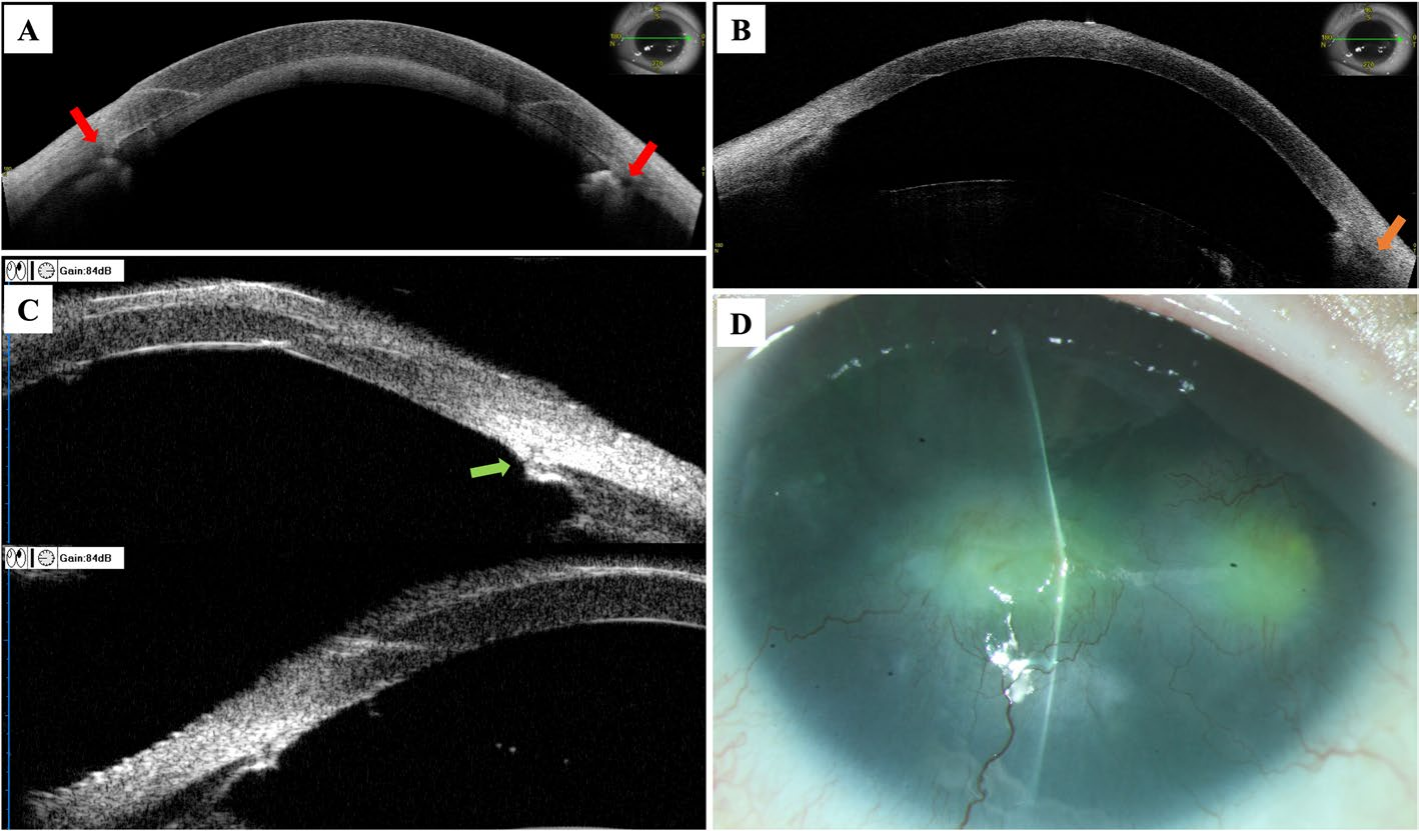
Postoperative examinations of the left eye. **A** Anterior segment optical coherence tomography 1-day postoperatively. **B** Anterior segment optical coherence tomography 1-year postoperatively. **C** The ultrasound biomicroscopy 1-year postoperatively. The arrowheads demonstrated the successful incision of the Schlemm canal. **D** The anterior segment photo was similar to that of preoperatively

At the first postoperative day examination, the IOP decreased to 10 mmHg, and Grade II hyphema was presented. Anterior segment optical coherence tomography (AS-OCT) showed successful incision of the Schlemm canal (Fig. 5).

At 6-month follow-up, the VA of left eye was 0.9 (logMAR) and the IOP was 22mmHg with topical brinzolamide/timolol BID.

One year post MAT surgery, the VA of left eye was 0.9 (logMAR) and the IOP was 19mmHg with topical latanoprost/timolol QN (XALACOM^®^, Pfizer, New York City, USA). The AS-OCT showed that the trabecular meshwork was still incised on the temporal side. The nasal iris stump adhered anteriorly with the angle wall (Fig. 5).

### Discussion and conclusions

According to the study of Gramer et al., congenital glaucoma is rare in aniridia patients, and the onset of glaucoma occurs in adolescence and early adulthood most [13]. Therefore, the monitoring of glaucoma should have been sustained since the diagnosis of aniridia. Surgery is often necessary to treat aniridic glaucoma. Different advocations about the methods of surgery reveal the refractoriness of aniridic glaucoma.

Various surgical procedures, including goniosurgeries, trabeculectomy, glaucoma drainage implants and cyclodestructive procedures, have been reported in previous researches. Goniosurgeries, including goniotomy and trabeculotomy, are the most reported approaches. The success rate of goniotomy varied in previous studies depending on the timing of intervention. Prophylactic goniotomy showed far better prognosis compared with therapeutic one [1]. Adachi et al. summarized 12 aniridic glaucoma eyes receiving trabeculotomy, and the results showed that 10 eyes received good IOP control with an average follow-up of 9.5 years. Thus, they suggested that trabeculotomy should be adopted as an initial method [12]. On the contrary, trabeculectomy demonstrates limited success rate. The study of Durai et al. revealed that the cumulative probability of failure was 58.3% in the trabeculectomy group at 2 years [14], and Wiggins et al. reported a success rate of only 7% receiving trabeculectomy [15]. Glaucoma drainage implant is another option. Almousa et al. implanted the Ahmed glaucoma valve for 8 aniridic glaucoma eyes, and 7 eyes received good IOP control [16]. Wiggins et al. also reported successful outcomes of glaucoma drainage implant in advanced patients who had already received prior glaucoma surgeries [15]. Although the efficacy of IOP control is good, complications related to implants, e.g., tube exposure, tube obstruction and corneal endothelial damage, may still occur in a high incidence [15–17]. Therefore, it may be better to preserve glaucoma drainage implants as a remedial procedure. Though cyclodestructive procedures are effective in controlling IOP, they often cause serious complications including hypopsia, cataract, retinal detachment and phthisis bulbi [15, 18, 19]. It may be unsuitable to select cyclodestructive surgery as an initial surgical choice to treat aniridic glaucoma patients who remain poor vision. The clinical information of prior studies is demonstrated in Table 1. In summary, prophylactic goniotomy provides good outcome in patients whose angle is open in early stage. However, surgical interventions demonstrate either limited efficacy or high incidence of postoperative complications in advanced patients. A new surgical approach is needed urgently for aniridic glaucoma patients following failed previous surgery.

**Table 1:** Summary of previous studies and current study on aniridic glaucoma patients treated with various surgical procedures

Surgical procedures	Cases	Number of enrollments (eyes)	Mean age at surgery (years)	Criteria for success	Average follow-up time (years)	Success rate (%)	Complications
**Prophylactic goniotomy**	Chen et al. (1999) [17]	55	3.1	IOP < 22mmHg w/o medications	9.5 [range, 0.7 ~ 24]	89.1	Not observed
**Therapeutic goniotomy**	Walton (1986) [20]	14	7	IOP ≤ 21mmHg w/ or w/o medications	3.8 [range, 0 ~ 11]^a^	21.4	Not observed
**Trabeculotomy**	Adachi et al. (1997) [12]	12	4.7	IOP ≤ 21mmHg w/ or w/o medications and no further glaucoma surgery	11.6 [range, 4 ~ 23]	83.3	Hyphema (8.3%) and transient IOP rise (8.3%)
**Trabeculectomy**	Durai et al. (2021) [14]	12	17.2	5mmHg < IOP ≤ 21mmHg or reduced ≥ 20% from baseline on 2 consecutive follow-up after 3 months, w/o glaucoma reoperation, complications and loss of light perception	3.6 [range, 2 ~ 4]	41.7 (at 2-year follow-up)^b^	Superior ciliary staphyloma (8.3%), retinal detachment (8.3%) and cataract (41.7%)
	Wiggins et al. (1992) [15]	15	N/A^c^	IOP ≤ 21mmHg w/ or w/o medications and no complications resulting in significant visual loss	N/A^c^	6.7	Retinal detachment (6.7%)
**Glaucoma drainage implants**	Almousa et al. (2013) [16]	8	49	5mmHg < IOP < 22mmHg w/ or w/o medications, w/o loss of light perception and no further glaucoma surgery	3.1 [range, 2.6 ~ 5.7]	87.5	Hyphema (12.5%), Persistent vitreous hemorrhage (12.5%), retinal detachment (12.5%) and phthisis bulbi (12.5%)
	Wiggins et al. (1992) [15]	6	N/A^c^	IOP ≤ 21mmHg w/ or w/o medications and no complications resulting in significant visual loss	N/A^c^	83.3	Tube migration (16.7%)
**Cyclodestructive procedures**	Wagle et al. (1998) [18]	8	N/A^c^	IOP ≤ 21mmHg w/o devastating complications or need for further glaucoma surgery	N/A^c^	25	Phthisis bulbi (50%) and retinal detachment (12.5%)
	Wallace et al. (1998) [19]	9	4.8	IOP ≤ 25mmHg w/ medications or better	11.9 [range, 4.5 ~ 17.9]	66.7	Cataract (77.8%)
	Wiggins et al. (1992) [15]	22	N/A^c^	IOP ≤ 21mmHg w/ or w/o medications and no complications resulting in significant visual loss	N/A^c^	22.7	Phthisis bulbi (9.1%) and progressive cataract (4.5%)
**Illuminated microcatheter-assisted circumferential trabeculotomy**	This case report	1	21	IOP ≤ 25mmHg w/ or w/o medications	1	N/A	Not observed

*N/A *Not Applicable, *IOP* Intraocular pressure, *w/o* Without, *w/* With

^a^In the study of Walton [20], 6 eyes received alternative procedures due to the unsuccessful IOP control immediately after therapeutic goniotomy. Therefore, the minimal follow-up period was recorded as ‘0’

^b^In the study of Durai et al. [14], the authors mentioned that the IOP was not significantly different from the preoperative level at 3-year and 4-year follow-up, and they only counted the success rate at 2-year follow-up

^c^In the studies of Wagle et al. [18] and Wiggins et al. [15], the authors did not count the mean age and average follow-up time in a specific procedure or aniridic glaucoma specifically. Therefore, we marked N/A in the corresponding blank

To the best of our knowledge, this case is unique in its first use of MAT for treating aniridic glaucoma. Our patient received goniotomy previously, but failed to control the IOP after 3 years. The result is in accordance with previous reports [1, 7]. We attempt to figure out a better approach with higher success rate and fewer complications. Adachi et al. advocated that abnormality of Schlemm canal, which was similar to congenital glaucoma, was a mechanism of aniridic glaucoma [12]. In several recent literatures, MAT showed satisfied results in patients with congenital glaucoma, and many of these cases possessed cloudy corneas. In these researches, the criteria of success were defined as IOP ≤ 21 mm Hg with or without antiglaucoma medications. The success rate reached 88% and no severe complications were observed [9, 21, 22]. Therefore, these researches provide theoretical basis for us to use MAT to treat aniridic glaucoma. The advantage of circumferential trabeculotomy is the ability to maximize the IOP-reducing effect by treating the whole anterior chamber angle in a single surgery. The illuminated catheter tip also enables us to see the location directly across the sclera without the need of direct gonioscopy which may need a certain extent of corneal clarity. The postoperative IOP remains stable generally with topical medications within 1 year in our patient. The application of MAT in the treatment of aniridic glaucoma may be regarded as qualified successful. Compared with goniotomy which shows higher success rate in younger patients who have not developed glaucoma yet, MAT may be a potential option for treating aniridic glaucoma patient who is older and has experienced a previously failed surgery.

In conclusion, we exhibited a possible surgical approach to manage *PAX6* aniridic glaucoma. However, further studies are needed to evaluate the efficacy and safety of MAT on aniridic glaucoma with more cases and longer follow-up period.

## Data Availability

The datasets generated and analyzed during the current study are available in the Genome Sequence Archive in National Genomics Data Center, China National Center for Bioinformation. The accession number is HRA006341 and the datasets can be accessed from the following web link: https://bigd.big.ac.cn/gsa-human/browse/HRA006341.

## Abbreviations

AS-OCT: Anterior segment optical coherence tomography
IOP: Intraocular pressure
MAT: Illuminated microcatheter-assisted circumferential trabeculotomy
*PAX6*: Paired box-containing mammalian gene 6
UBM: Ultrasound biomicroscopy
WAGR: Wilms tumor, aniridia, genitourinary anomalies and mental retardation
*WT1*: Wilms tumor predisposition gene
